# Application of Raman spectroscopy in type 2 diabetes screening in blood using leucine and isoleucine amino-acids as biomarkers and in comparative anti-diabetic drugs efficacy studies

**DOI:** 10.1371/journal.pone.0185130

**Published:** 2017-09-19

**Authors:** Zephania Birech, Peter Waweru Mwangi, Fredrick Bukachi, Keith Makori Mandela

**Affiliations:** 1 Department of Physics, University of Nairobi, P.O Box 30197-00100, Nairobi, Kenya; 2 Department of Medical Physiology, University of Nairobi, P.O Box 30197-00100, Nairobi, Kenya; Technische Universitat Munchen, GERMANY

## Abstract

Diabetes is an irreversible condition characterized by elevated blood glucose levels. Currently, there are no predictive biomarkers for this disease and the existing ones such as hemoglobin A1c and fasting blood glucose are used only when diabetes symptoms are noticed. The objective of this work was first to explore the potential of leucine and isoleucine amino acids as diabetes type 2 biomarkers using their Raman spectroscopic signatures. Secondly, we wanted to explore whether Raman spectroscopy can be applied in comparative efficacy studies between commercially available anti-diabetic drug pioglitazone and the locally used anti-diabetic herbal extract *Momordica spinosa* (*Gilg*.)*Chiov*. Sprague Dawley (SD) rat’s blood was used and were pipetted onto Raman substrates prepared from conductive silver paste smeared glass slides. Prominent Raman bands associated with glucose (926, 1302, 1125 cm^−1^), leucine (1106, 1248, 1302, 1395 cm^−1^) and isolecucine (1108, 1248, 1437 and 1585 cm^−1^) were observed. The Raman bands centered at 1125 cm^−1^, 1395 cm^−1^ and 1437 cm^−1^ associated respectively to glucose, leucine and isoleucine were chosen as biomarker Raman peaks for diabetes type 2. These Raman bands displayed decreased intensities in blood from diabetic SD rats administered antidiabetic drugs pioglitazone and herbal extract *Momordica spinosa* (*Gilg*.)*Chiov*. The intensity decrease indicated reduced concentration levels of the respective biomarker molecules: glucose (1125 cm^−1^), leucine (1395 cm^−1^) and isoleucine (1437 cm^−1^) in blood. The results displayed the power and potential of Raman spectroscopy in rapid (10 seconds) diabetes and pre-diabetes screening in blood (human or rat’s) with not only glucose acting as a biomarker but also leucine and isoleucine amino-acids where intensities of respectively assigned bands act as references. It also showed that using Raman spectroscopic signatures of the chosen biomarkers, the method can be an alternative for performing comparative efficacy studies between known and new anti-diabetic drugs. Reports on use of Raman spectroscopy in type 2 diabetes mellitus screening with Raman bands associated with leucine and isoleucine molecules acting as reference is rare in literature. The use of Raman spectroscopy in pre-diabetes screening of blood for changes in levels of leucine and isoleucine amino acids is particularly interesting as once elevated levels are noticed, necessary interventions to prevent diabetes development can be initiated.

## 1 Introduction

Diabetes is a disease that is increasingly affecting many people around the world. According to the World Health Organization (WHO) by the year 2014, there were 422 million people living with diabetes [1]. This figure was four times greater than that reported in 1980 which stood at around 108 million. The disease is characterized by increased levels of blood glucose due to malfunctioned production of insulin [2–4]. There are two types of diabetes: type 1 diabetes and type 2 diabetes mellitus [1]. The former is common among children and the cause not known while the later affects majority of people and result largely from excess body weight and less physical activity [1, 2, 5]. The disease causes many complications including renal failure, blindness, nerve damage and vascular disease.

The standard method of diabetes diagnosis is through measurement of blood glucose levels in a blood drop placed on a diagnostic strip [2]. The enzymatic reaction of the sample in the strip with glucose oxidate results in formation of glucomic acid and hydrogen peroxide [6]. The change in current between electrodes on the test strip is then read out [6, 7]. This so called fasting plasma glucose (FPG) measurement suffers some disadvantages in that one has to fast and then blood drawn, besides having low sensitivity (40–60%) [7]. The other diabetes screening alternative involves monitoring of hemoglobin variation [8]. Hemoglobin in diabetic patients can vary as a result of the excess sugar in blood getting bonded with hemoglobin (protein) molecules without enzymatic regulation (process known as hemoglobin glycation) [5, 8, 9]. This later method requires use of external dyes or reagents and is ideal for long term diabetes monitoring (about three months, the lifespan of red blood cells) [9].

There have been efforts to develop other alternative quick diabetes screening techniques lately. Most of them are optical based with Raman and infrared spectroscopy being prominent due to their potential for rapid, label-free, non-invasive and in-vivo diabetes screening applications. The conventional Raman spectroscopy where no special sample substrate is required has been used in measurement of glucose (sugar) concentration in blood where intensities of the characteristic spectral bands and spectral profiles are used in quantification and discrimination between diabetic and non-diabetic patients [3, 4, 6, 10]. Prospects of the technique in in-vivo glucose measurements [4] and device miniaturization for diabetes screening [6] have also been reported. The sensitive variant of the technique known as Surface-enhanced Raman spectroscopy (SERS) has been shown to detect glucose of concentrations between 1–5 *μ*M at the glucose signature spectral band centered at 986 cm^−1^ [11]. SERS has also been shown to distinguish between concentration of oxyhemoglobin in patients suffering from diabetes type 2 and those non-diabetic [8]. In SERS, specially prepared roughened metallic (silver, gold, copper etc.) sample substrates or metallic colloids are needed.

Diabetic condition is so far irreversible and only managed through approriate medicines and special diet. Alot of work is on-going on identification of predictive biomarkers of this condition. Amino acids, in particular the branched-chain amino acids (BCAAs) which includes leucine, isoleucine and valine are increasingly becoming potential canditate biomarkers for predicting risk of type 2 diabetes [12–14]. Several studies show that BCAAs are elevated in plasma and urine of obese, insulin-resistant and type 2 diabetic subjects [12–14]. Among BCAAs, leucine is th most abundant in many dietary proteins [14]. It has been shown that BCAA concentration level monitoring can predict onset of type 2 diabetes as early as four years [15]. At present there are no confirmed predictive diabetes biomarkers and the existing ones such as hemoglobin A1c and fasting glucose only diagnose overt diabetes [12]

In this work we explored two things (i) the potential of leucine and isoleucine amino acids as diabetes type 2 biomarkers using their Raman spectroscopic signatures and (ii) whether Raman spectroscopy can be applied in comparative efficacy studies between commercially available anti-diabetic drug pioglitazone and the locally used anti-diabetic herbal extract *Momordica spinosa* (*Gilg*.)*Chiov*. Sprague Dawley (SD) rats were chosen for the study due to their ease of handling and calmness. The blood drawn from these rats were first pipetted onto Raman substrates prepared from conductive silver paste smeared glass slides. A similar substrate was shown recently to be an excellent Raman substrate in Raman spectroscopic screening of blood plasma for human immuno-deficiency virus (HIV) [16]. With our substrate, Raman spectroscopy clearly discriminated between diabetic and non-diabetic SD rats based on Raman spectral bands ascribed to both glucose and the two branched-chain amino acids (BCAA) leucine and isoleucine. The use of Raman spectroscopy in diabetes screening based on concentration of BCAA was not found in literature by the time of preparing this manuscript. We also showed that the technique can be used in monitoring performance of anti-diabetic drug pioglitazone in comparison with local herbalist’s anti-diabetic drug *Momordica spinosa* (*Gilg*.)*Chiov*. In the comparative anti-diabetic performance study, intensity of Raman peaks associated with glucose, leucine and isoleucine were used as reference. The plant *Momordica spinosa* (*Gilg*.)*Chiov* which is indigenous to Kenya, but also found in Somalia and Ethiopia is traditionally used by herbalists in diabetes and respiratory disease treatment [17, 18]. Pioglitazone on the other hand is a common commercially available anti-diabetic drug often used along with proper diet and exercise in order to control blood sugar in patients with type 2 diabetes mellitus [19, 20].

## 2 Experimental

In the study, 40 freshly weaned four week old Sprague Dawley (SD) rats were used. The sample size was calculated using the relation $sample\;size=\frac{2{\left(SD\right)}^{2}{\left(Z_{\alpha /2}+Z_{\beta }\right)}^{2}}{d^{2}}$ as described by Jaykaran C. and Tamoghna B. in [21]. In the formula, *SD*, *Z*_*α*/2_, *Z*_*β*_ and *d* represent the standard deviation from previous study or pilot, standard normal variate for level of significance, standard normal variate for power and effect size respectively. The rats were housed, 5 members each, in metallic cages of dimensions 109 cm by 69 cm by 77.5 cm with floor covered with wood shavings replaced thrice every week. Lighting was maintained at a 12 hour day and night cycle. The animals were habituated for 7 days before grouping them into four of ten members each for purposes of feeding with different food diets. Before commencement of the feeding programme, the rats were first fasted for five hours, each separately anaesthetized in an air-tight desiccator using halothane soaked on cotton wool. A rat would be withdrawn from the desiccator once it lay on its side with slowed breathing. Anesthesia would be confirmed by checking for loss of the blink reflex. Blood (100 *μ* l per rat) was then collected by lateral tail vein sampling and was stored in sodium citrate vacutainers to prevent clotting and refrigerated at 4°C. The diabetes negative status (Normal/non-diabetic) of the 40 rats were then first confirmed through Fasting blood glucose (FBG) measurement using a commercial glucometer (GlucoPlus Inc 2323 Halpern Ville St-Laurent Quebec Canada GPMS 214823). At the end of the experimental period, all the rats were euthanized after an overnight fast using an intraperitoneal injection of 20% Phentobarbital (1ml/kg of body weight). Death was confirmed by loss of the pupillary light reflex.

Fresh whole plants of *Momordica spinosa* (*Gilg*.)*Chiov* were collected from their natural habitat in Machakos county in Kenya and their identity was confirmed at the University of Nairobi herbarium and a voucher specimen deposited therein. The plants were air dried for a week before being ground into a dry powder. This powder (1 kg) was macerated in distilled water for 20 minutes in a weight: volume ratio of 1:8, producing 8 litres of solution. This solution was then filtered in two stages. Coarse filtration was done through cotton wool, and fine filtration of the resulting filtrate was then done through filter paper. The filtrate was frozen before undergoing lyophilization to obtain the freeze-dried extract. The extract was stored in the deep freezer at the Department of Medical Physiology covered with parafilm to prevent entry of moisture. The high and low dose solutions were prepared with respect to the weight of the rats in the experimental groups and administered daily by oral gavage on an individualized weight-specific basis.

After diabetes negative status confirmation, all the animals were then injected intraperitoneally on Day 29 with low dose alloxan (40 mg/kg of body weight) to induce type 2 diabetes mellitus. Alloxan is a toxic chemical that is known to destroy insulin producing cells (beta cells) in the pancreas as observed in rodents and other animal species [22, 23]. The SD rats were thereafter fed on different diets after grouping them into four of 10 members each. The diet used were as follows (i) high fat and high sugar diet only (Diabetic), (ii) high fat and high sugar diet plus 200mg/kg body weight of *Momordica spinosa* (*Gilg*.)*Chiov* herb extract (MS200) (iii) high fat and high sugar diet plus 400mg/kg body weight of *Momordica spinosa* (*Gilg*.)*Chiov* herb extract (MS400) and (iv) high fat and high sugar diet plus Pioglitazone, 20mg/kg of body weight (P). The *Momordica spinosa* (*Gilg*.)*Chiov* extracts and Pioglitazone were administered throughout the study period by daily oral gavage. All groups were fed on a high fat diet (20%) and high sugar diet (30% sucrose) ad libitum for six weeks. The high fat diet was prepared by adding 45g of cooking fat to 225g of standard chow pellets (Unga Feeds Limited, Nairobi). The mixture was gently heated over low heat for 30 minutes while stirring. 30 g of sugar (99% sucrose) was dissolved in 100ml water to achieve 30% sucrose solution. The study was conducted in two phases. In the first phase (which lasted for 28 days), the protective effects of *Momordica spinosa* (*Gilg*.)*Chiov* herb extract against diet-induced diabetes were investigated. In the second phase (day 29 to day 42), the protective effects of *Momordica spinosa* (*Gilg*.)*Chiov* herb extract against drug-induced diabetes (induced by an intraperitoneal injection of Alloxan) were investigated.

**Fig 1:**
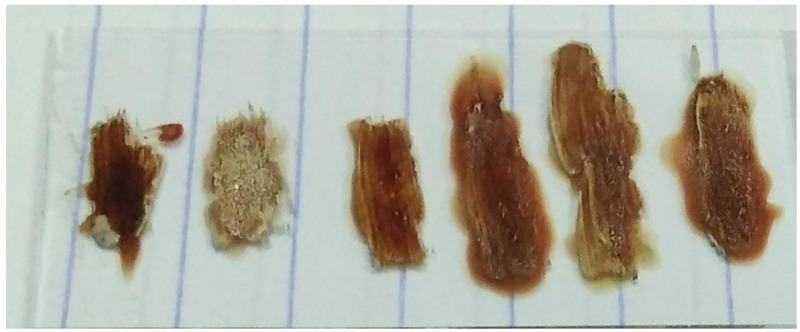
Figure showing image of SD rat’s blood (non-diabetic) applied onto the conductive silver paste smeared glass slide. Patches of the smear were made as shown.

The Raman sample substrates were made from smearing conductive silver paste/paint (SPI supplies, USA) on microscope slides using a brush and air dried for 30 minutes. Each patch smear contained ≈300 *μ*L of the paste and were of dimensions of ≈10 mm by ≈20 mm by ≈0.5 mm. Several smear patches were made on one microscope glass slide as shown in Fig 1. The conductive silver paste composition included silver nanoparticles: 35–60% per weight; 1-methoxy-2 propanol acetate: 10–30% weight; batyl acetate: 10–30% weight and acrylic resin: 5–10% weight. The blood(≈100 *μ* L) drawn from the five hour fasted Sprague Dawley (SD) rats were then pipetted onto the silver paste smeared glass slides and let dry for one hour. For Raman spectroscopy, confocal laser Raman microscope system (STR, Seki Technotron Corp) equipped with a 785 nm laser and a spectrometer (Princeton Instruments) was used. The experimental parameters used were: excitation wavelength, 785 nm; excitation power at sample position, ≈1*μ*W; beam spot size at sample position, ≈40 *μ*m; spectra accumulation, 10; exposure time, 10 s; x50 microscope objective (Max Plan) with 0.50 numerical aperture. A 600 lines per millimeter grating was chosen in order to cover a wider spectral region. The spectrometer was calibrated using the strong Raman peak of the silicon wafer centered at 520 cm^−1^ before every experimental session. Five spectra from five different random spots in each sample were collected. The obtained Raman spectral data were first smoothened and auto-fluorescence background removed using Vancouver Raman Algorithm based on fifth-order polynomial fitting method developed by Zhao *et al* [24]. The data were thereafter normalized by subtracting the minimum intensity value in each data set and then plotted using ORIGIN 2015 software. This analysis was done within the ‘finger print’ spectral region 400–1800 cm^−1^ centered at 1055.85 cm^−1^. Both the blood and BCAA crystal samples were excited with silver paste smeared glass slide as the substrate in order to eliminate fluorescence background (usually centered around 1100 cm^−1^) emanating from the glass slide.

## 3 Results and discussion

**Fig 2:**
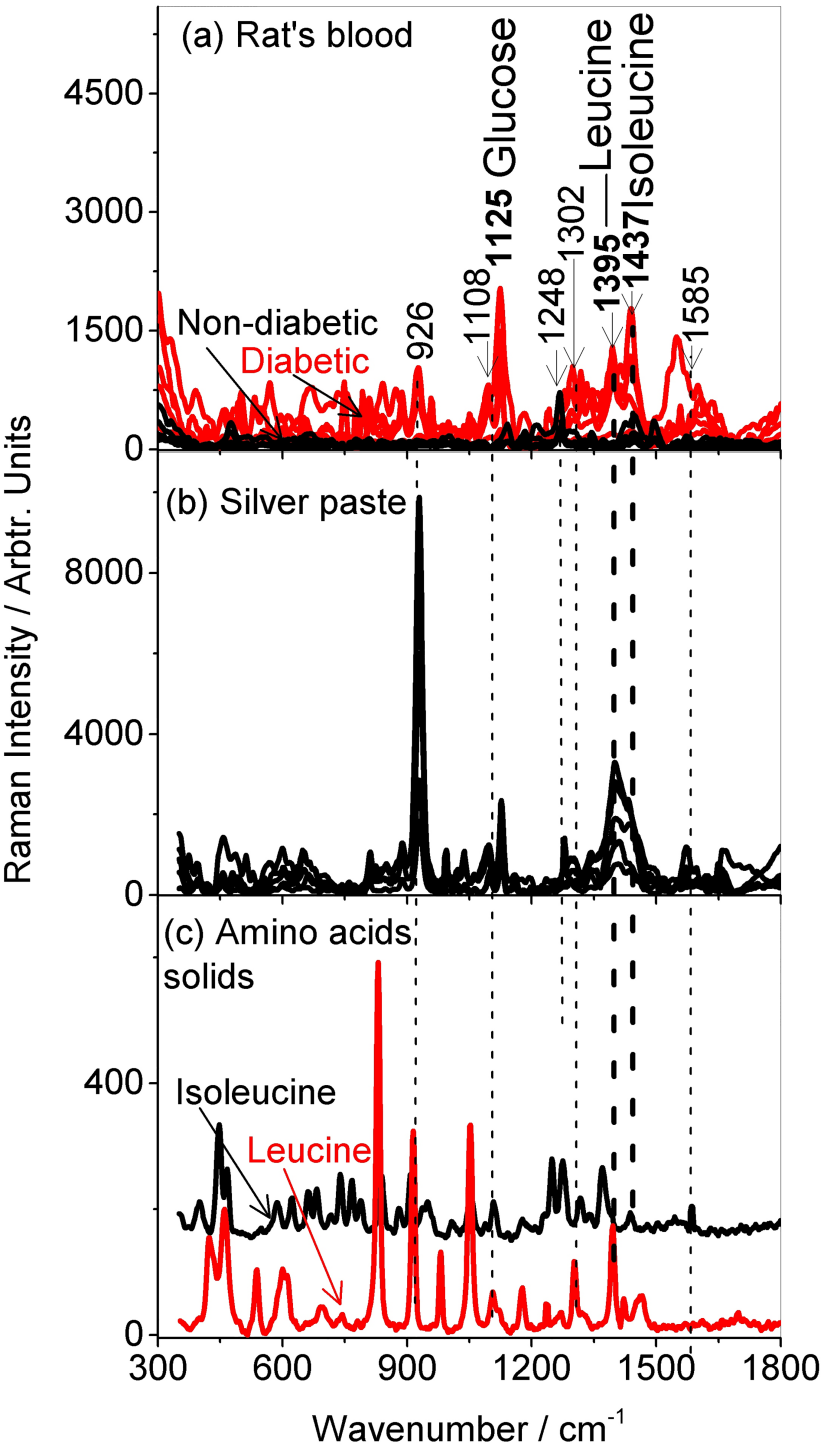
Figure showing the Raman spectra of (a) SD rats both diabetic and non-diabetic (b) conductive silver paste smear on glass slide and (c) branched-chain amino acid solids leucine and isoleucine after 785 nm excitation at room temperature. Five spectra each are displayed in (a-b) and and average of five in (c).

The Raman spectra obtained from blood applied onto the silver paste smeared glass slides for both diabetic and non-diabetic rats are displayed in Fig 2. The spectra of silver paste smear and the branched-chain amino acids (BCAA) leucine and isoleucine solids are also shown. Application of the silver paste smear was observed to suppress the usually broad fluorescence band centered around 1100 cm^−1^ associated with microscope glass slides upon 785 nm laser excitation. The blood from diabetic rats displayed generally intense Raman bands compared to those from non-diabetic rats. The exact reason for the intensity enhancement was not known but thought to be due to various increase in other Raman active protein molecules whose Raman signals get enhanced with presence of silver paste smear contents. The prominent bands were those associated with glucose: 926 cm^−1^ assigned to (C-O) and (C-C) stretching vibration [4, 6], 1108 cm^−1^ and 1125 cm^−1^ assigned to (C-OH) and (C-O-H) stretching vibrations [6]; branched-chain amino acids (BCAA): leucine (1106, 1248, 1302 and 1395 cm^−1^) [25–27] and isolecucine (1108, 1248, 1437 and 1585 cm^−1^) [25, 28]with vibrational mode assignments given in Table 1. The bands centered at 926, 1125 cm^−1^ (glucose), 1395 cm^−1^ (leucine) and 1437 cm^−1^ (isoleucine) overlapping intense conductive silver paste bands were enhanced (see Fig 2). This intensity enhancement can be attributed to the respective molecules in blood (glucose, leucine and isoleucine) getting attached to the silver nanoparticles (35–60% per weight) in the paste and on interaction with the laser resulted in plasmon resonance enhancement of their Raman signals. The other constituents of the paste (1-methoxy-2 propanol acetate: 10–30% weight; batyl acetate: 10–30% weight; acrylic resin: 5–10% weight) might have also played a role especially in as far as glucose molecules are concerned. It was also noticed that some of the intense peaks seen in the BCAA crystal samples such as leucine’s bands centererd at 834 cm^−1^ and 1050 cm^−1^ were suppressed in the blood samples. This may be due to several surrounding molecules in the complex (i.e. blood and conductive silver paste smear) influencing their bonds and hence the respective Raman bands.

**Table 1:** Table showing the center wavenumbers of the prominent Raman bands in the spectra from mainly diabetic SD rat’s blood, leucine and isoleucine amino acids and their tentative assignments.

Leucine (cm^−1^)	Isoleucine(cm^−1^)	Diabetic Rat’s blood(cm^−1^)	Tentative assignment
913	907	-	(C-C), (C-N) stretching in leucine and isoleucine [25]
-	-	926	(C-O) and (C-C) stretch in glucose [4, 6]
1106	1108	1108	(C-C), (C-N) stretching in leucine and isoleucine [25], (C-OH) and (C-O-H) stretch in glucose [6]
-	-	1125	(C-OH) and (C-O-H) stretch in glucose [6, 10]
1236	1248	1248	(CH_2_)Torsion in leucine and isoleucine [25, 29], (C-O-H)deformation in leucine [26]
1302	-	1302	(CH) deformation in leucine
1395	-	1395	(CH) (CH_3_) bending [25], (CH_3_) deformation in leucine [26, 27]
-	1437	1437	Assymetric rocking, symmetric bending of C atoms in isoleucine [25, 28]
-	1585	1585	

It is known that glucose has weak Raman signals and poor adsorbidity onto metals [11] making surface enhanced Raman spectroscopy less sensitive to it. The constituents in the silver paste may have acted as a link between glucose molecules and the silver nanoparticles in the paste smeared glass slides in a similar manner as 2-Thienylboronic acid (2-TBA) did elsewhere [11]. The intense Raman peaks in diabetic rat’s blood also indicated elevated concentrations of the respective molecules (glucose, leucine and isoleuine). The role of BCAA levels change in blood plasma and urine as potential metabolic disease biomarkers has been generating research interest [30, 31]. Elevated levels of these molecules (i.e. BCAA) in blood has been associated with obesity, insulin resistance and type 2 diabetes [13–15]. Since Raman scattering cross-section increases when a molecule of the analyte comes in contact with a metallic nanoparticle [32], an increased concentration of these molecules on the silver paste smeared glass substrate should result equally to intense Raman bands associated with it. Raman intensity is, therefore, related to concentration and it can be interpreted that levels of leucine and isoleucine in diabetic SD rats studied here were higher than in non-diabetic ones as expected [13–15]. Measurement of BCAAs is conventionally done using high performance liguid chromatography (HPLC) [33] which involves use of reagents and is time consuming. The results obtained here demonstrated the potential of Raman spectroscopy in rapid (10 seconds) diabetes or pre-diabetes screening in humans with leucine (1395 cm^−1^) and isoleucine (1437 cm^−1^) Raman peak intensities acting as reference. Pre-diabetes detection via Raman spectroscopic screening of blood for elevated changes in levels of leucine and isoleucine amino acids can provide an opportunity of preventing development of diabetes as appropriate interventions can be initiated. Most patients are usually confirmed to be diabetic when it is already too late to reverse the condition as only tests are initiated once symptoms such as frequent urination, excessive thirst, increased hunger, weight loss, tiredness etc. presents.

**Fig 3:**
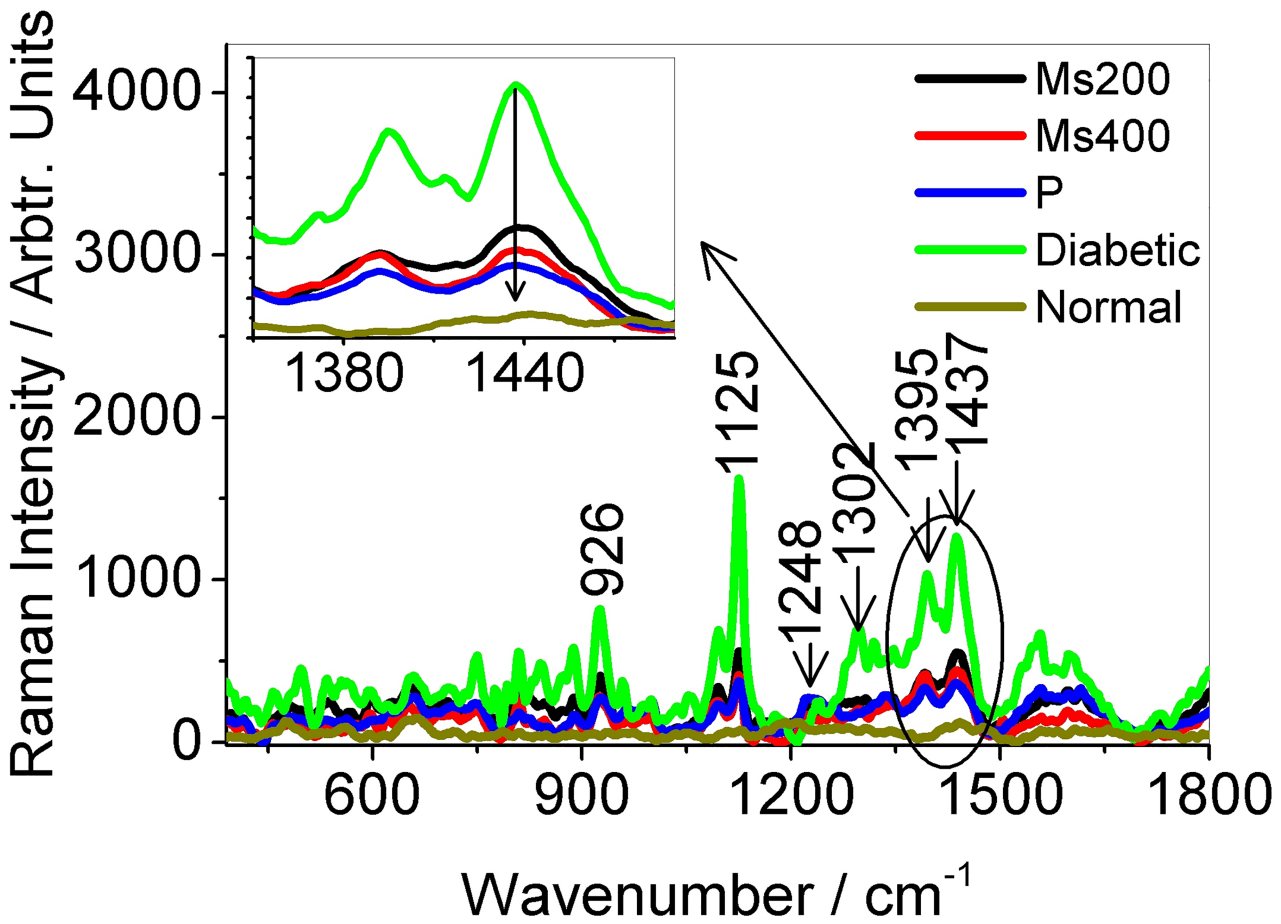
Figure showing the avarage Raman spectra (average of five spectra each) from diabetic rats given anti-diabetic drug pioglitazone (P), herbal extracts *Momordica spinosa* (*Gilg*.)*Chiov*. at concentrations of 200 mg/ kg (Ms200) and 400 mg/kg (Ms400) of body weight. Spectra from non-diabetic rats (Normal) is also given. The inset shows the changes in intensity at peaks centered at wavenumbers 1437 cm and 1395 cm^−1^.

**Fig 4:**
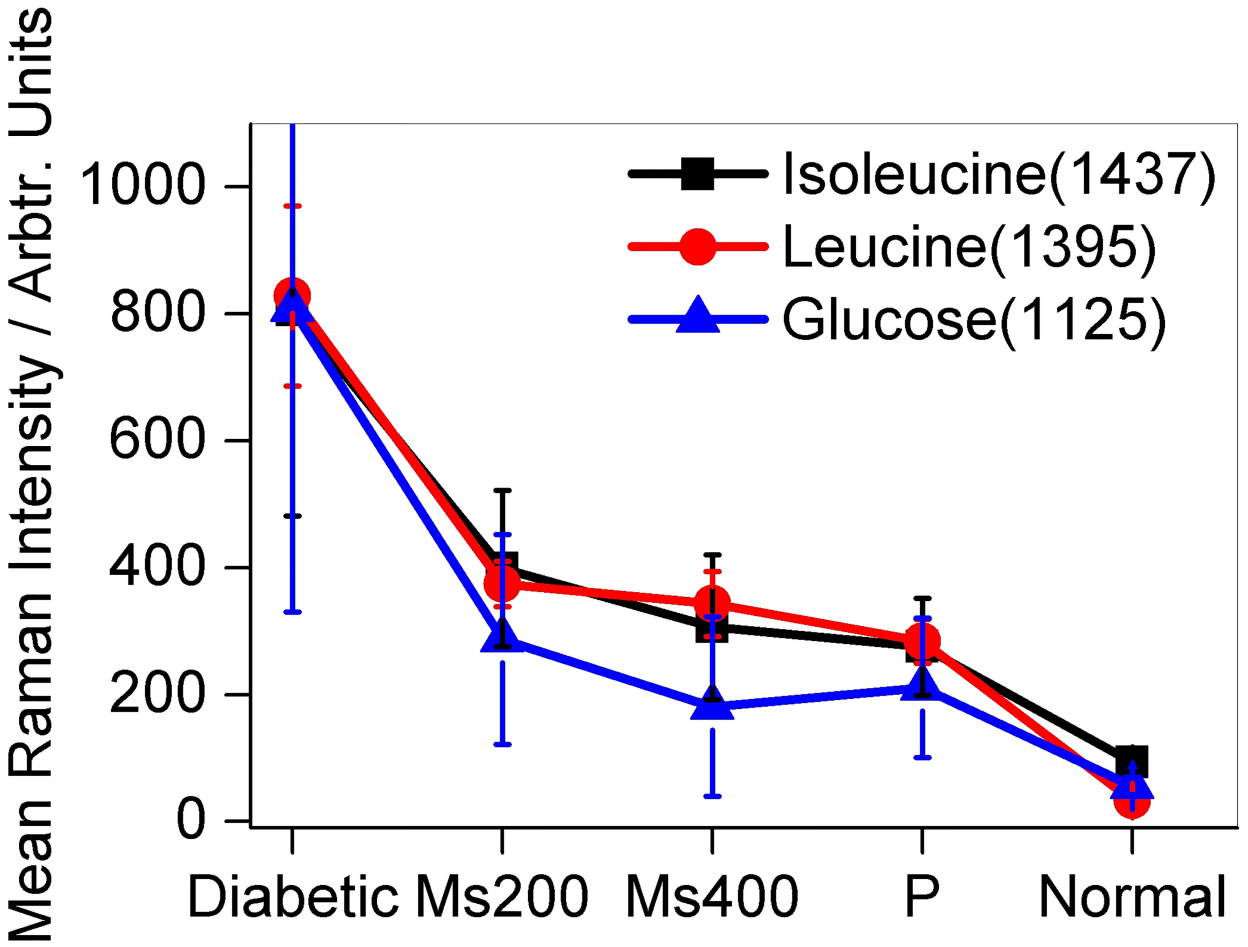
Figure showing the changes in the mean Raman intensity of diabetic SD rat’s blood at bands centered at wavenumbers 1125, 1395 and 1437 cm^−1^ assigned to glucose, leucine and isoleucine respectively after giving them anti-diabetic drugs pioglitazone and herbal extracts at concentrations of 200 mg/kg (Ms200) and 400 mg/kg (Ms400) of body weight. The intensities decreased with administration of anti-diabetic drugs and extracts.

To further show that the intensities of Raman peaks associated with glucose, leucine and isoleucine can be used for diabetes screening, blood from diabetic rats given anti-diabetic drug pioglitazone and traditional anti-diabetic herbal extracts *Momordica spinosa* (*Gilg*.)*Chiov*. were also investigated. The results are displayed in Figs 3 and 4. The mean Raman intensities at peaks centered at wavenumbers 1125, 1395 and 1437 cm^−1^ assigned to glucose, leucine and isoleucine respectively decreased when the SD rats were given the anti-diabetic drug pioglitazone and herbal extracts (see Fig 4). The reduction in intensities at the bands of interest as a result of administration of anti-diabetic drugs can be interpreted as arising due to decreased concentration of glucose, leucine and isoleucine molecules in blood samples under study. Based on this interpretation, Fig 4 also tell us that the concentration of glucose molecule in diabetic SD rats are more affected by administration of the anti-diabetic drugs than the two BCAAs (leucine and isoleucine) though all displayed a decreasing trend. It can also be noticed that the herbal extract *Momordica spinosa* (*Gilg*.)*Chiov* at a concentration of 400mg/kg of SD rats body weight had more effect on glucose concentration than pioglitazone. This showed the great potential of the traditional extract in diabetes management. The results also indicated that Raman spectroscopic method with the blood samples applied onto the silver smeared glass slides can be used in performing comparative anti-diabetic drug efficacy studies. Further reports on anti-diabetic activity of this herb (*Momordica spinosa* (*Gilg*.)*Chiov*) will be in subsequent publications. Also to be included are glucose, weight measurement and insulin tolerance test and lipid profile results that were carried out within the first phase (day 1 to day 28).

## 4 Conclusion

The above results display the great potential of Raman spectroscopy together with conductive silver paste smeared glass slides in Raman spectroscopic detection of diabetes and also in investigating effects of new drugs on diabetes in comparison with existing ones. The results also shows that in screening for diabetes or pre-diabetic condition, the changes in intensities of Raman bands associated with leucine and isoleucine amino acids can be used. The use of Raman spectroscopy in pre-diabetes screening of blood via monitoring changes in levels of leucine and isoleucine amino acids is particularly interesting as once elevated levels are noticed, necessary interventions to prevent diabetes development can be initiated. Raman spectroscopic screening of diabetes can be done in-vivo and the device has potential of being made portable hence making it interesting for quick screening in a large population non-invasively.

## 5 Compliance with ethical standards

Ethical approval for the study was granted by the Biosafety, Animal Care and Use Committee, Faculty of Veterinary Physiology, University of Nairobi.

## Supporting information

S1 File The ARRIVE guidelines checklist. (PDF)
