# Supplementary material for: Application of Raman spectroscopy in type 2 diabetes screening in blood using leucine and isoleucine amino-acids as biomarkers and in comparative anti-diabetic drugs efficacy studies
Source: PLoS One. 2017 Sep 19;12(9):e0185130. doi: 10.1371/journal.pone.0185130 (PMC5605051; doi:10.1371/journal.pone.0185130)
Supplement: S1 File — (PDF) [file pone.0185130.s001.pdf]

ARRIVE

The ARRIVE Guidelines Checklist

Application of Raman Spectroscopy in Type 2 Diabetes Screening in Blood Using Leucine and Isoleucine Amino-acids as Biomarkers and in Comparative Anti-diabetic Drugs Efficacy Studies

Zephania Birech[1], Peter Waweru Mwangi[2], Fredrick Bukachi[2] and Keith Makori Mandela[2]

[1]The Department of physics, University of Nairobi, P.O Box 30197-00100, Nairobi, Kenya, [2]School Department of medical Physiology, University of Nairobi, P.O Box 30197-00100, Nairobi, Kenya.

	ITEM	RECOMMENDATION	Section/ Paragraph
Title	1	Provide as accurate and concise a description of the content of the article as possible.	"Title"|
Abstract	2	Provide an accurate summary of the background, research objectives, including details of the species or strain of animal used, key methods, principal findings and conclusions of the study.	"Abstract"
**INTRODUCTION**			
Background	3	a. Include sufficient scientific background (including relevant references to previous work) to understand the motivation and context for the study, and explain the experimental approach and rationale.<br>b. Explain how and why the animal species and model being used can address the scientific objectives and, where appropriate, the study's relevance to human biology.	Section 1 "introduction " / Line 1-50<br>Line 55
Objectives	4	Clearly describe the primary and any secondary objectives of the study, or specific hypotheses being tested.	"Abstract", "introduction "/ Line 51-74
**METHODS**			
Ethical statement	5	Indicate the nature of the ethical review permissions, relevant licences (e.g. Animal [Scientific Procedures] Act 1986), and national or institutional guidelines for the care and use of animals, that cover the research.	Section 5 line 253-254
Study design	6	For each experiment, give brief details of the study design including:<br>a. The number of experimental and control groups.<br>b. Any steps taken to minimise the effects of subjective bias when allocating animals to treatment (e.g. randomisation procedure) and when assessing results (e.g. if done, describe who was blinded and when).<br>c. The experimental unit (e.g. a single animal, group or cage of animals).<br>A time-line diagram or flow chart can be useful to illustrate how complex study designs were carried out.	Section 2 "Experiment al" line 75-159.
Experimental procedures	7	For each experiment and each experimental group, including controls, provide precise details of all procedures carried out. For example:<br>a. How (e.g. drug formulation and dose, site and route of administration, anaesthesia and analgesia used [including monitoring], surgical procedure, method of euthanasia). Provide details of any specialist equipment used, including supplier(s).<br>b. When (e.g. time of day).<br>c. Where (e.g. home cage, laboratory, water maze).<br>d. Why (e.g. rationale for choice of specific anaesthetic, route of administration, drug dose used).	Section 2 "Experiment al" line 75-159.
Experimental animals	8	a. Provide details of the animals used, including species, strain, sex, developmental stage (e.g. mean or median age plus age range) and weight (e.g. mean or median weight plus weight range).<br>b. Provide further relevant information such as the source of animals, international strain nomenclature, genetic modification status (e.g. knock-out or transgenic), genotype, health/immune status, drug or test naïve, previous procedures, etc.	Section 2 "Experiment al" line 75-159.

Housing and husbandry	9	Provide details of:<br>a. Housing (type of facility e.g. specific pathogen free [SPF]; type of cage or housing; bedding material; number of cage companions; tank shape and material etc. for fish).<br>b. Husbandry conditions (e.g. breeding programme, light/dark cycle, temperature, quality of water etc for fish, type of food, access to food and water, environmental enrichment).<br>c. Welfare-related assessments and interventions that were carried out prior to, during, or after the experiment.	Section 2 "Experimental" line 75-159.
Sample size	10	a. Specify the total number of animals used in each experiment, and the number of animals in each experimental group.<br>b. Explain how the number of animals was arrived at. Provide details of any sample size calculation used.<br>c. Indicate the number of independent replications of each experiment, if relevant.	Section 2 "Experimental" line 75-159.
Allocating animals to experimental groups	11	a. Give full details of how animals were allocated to experimental groups, including randomisation or matching if done.<br>b. Describe the order in which the animals in the different experimental groups were treated and assessed.	Section 2 "Experimental" line 75-159.
Experimental outcomes	12	Clearly define the primary and secondary experimental outcomes assessed (e.g. cell death, molecular markers, behavioural changes).	Section 3 "results and discussion" Line 160-238
Statistical methods	13	a. Provide details of the statistical methods used for each analysis.<br>b. Specify the unit of analysis for each dataset (e.g. single animal, group of animals, single neuron).<br>c. Describe any methods used to assess whether the data met the assumptions of the statistical approach.	Section 3 "results and discussion" Line 160-238
RESULTS			
Baseline data	14	For each experimental group, report relevant characteristics and health status of animals (e.g. weight, microbiological status, and drug or test naïve) prior to treatment or testing. (This information can often be tabulated).	Section 3 "results and discussion" Line 160-238
Numbers analysed	15	a. Report the number of animals in each group included in each analysis. Report absolute numbers (e.g. 10/20, not 50%[2]).<br>b. If any animals or data were not included in the analysis, explain why.	Section 2 "Experimental" line 75-159.
Outcomes and estimation	16	Report the results for each analysis carried out, with a measure of precision (e.g. standard error or confidence interval).	Section 3 "results and discussion" Line 160-238
Adverse events	17	a. Give details of all important adverse events in each experimental group.<br>b. Describe any modifications to the experimental protocols made to reduce adverse events.	Section 2 "Experimental" line 75-159.
DISCUSSION			
Interpretation/ scientific implications	18	a. Interpret the results, taking into account the study objectives and hypotheses, current theory and other relevant studies in the literature.<br>b. Comment on the study limitations including any potential sources of bias, any limitations of the animal model, and the imprecision associated with the results[2].<br>c. Describe any implications of your experimental methods or findings for the replacement, refinement or reduction (the 3Rs) of the use of animals in research.	Section 3 "results and discussion" Line 160-238

Generalisability/ translation	19	Comment on whether, and how, the findings of this study are likely to translate to other species or systems, including any relevance to human biology.	Section 3 “results and discussion” Line 160-238
Funding	20	List all funding sources (including grant number) and the role of the funder(s) in the study.	Section 6 line 256-257

1. WHO, et al. Global report on diabetes. 2016;.
2. Šťovíčková L, Tatarkovič M, Logerová H, Vavřinec J, Setnička V. Identification of spectral biomarkers for type 1 diabetes mellitus using the combination of chiroptical and vibrational spectroscopy. Analyst. 2015;140(7):2266–2272.
3. Firdous S, Nawaz M, Ahmed M, Anwar S, Rehman A, Rashid R, et al. Measurement of diabetic sugar concentration in human blood using Raman spectroscopy. Laser Phys. 2012;22(6):1090–1094.
4. Li X, Li Y, Liang J, Lin M, Liu J, Shao J, et al. In Vivo Blood Glucose Quantification Using Raman Spectroscopy. PLoS One. 2012;7(10):1–6.
5. Lin J, Lin J, Huang Z, Lu P, Wang J, Wang X, et al. Raman spectroscopy of human hemoglobin for diabetes detection. J Innovative Opt Health Sci. 2014;7(01):1350051.
6. Wróbel M. Non-invasive blood glucose monitoring with Raman spectroscopy: prospects for device miniaturization. In: IOP Conf. Ser.: Mater. Sci. Eng.. vol. 104. IOP Publishing; 2016. p. 012036.
7. John M D, Mark R, Jeffrey W F, Catriona N M, Marwood E N. Noninvasive Type 2 Diabetes Screening: Superior sensitivity to fasting plasma glucose and A1C. Diabetes Care. 2007;30:1120–1124.
8. Lin J, Huang Z, Feng S, Lin J, Liu N, Wang J, et al. Label-free optical detection of type II diabetes based on surface-enhanced Raman spectroscopy and multivariate analysis. J Raman Spectrosc. 2014;45(10):884–889.
9. Ishan B, Narahara D Chari, Jeon K Woong, Gary H L, Ramachandra D R, Michael F S. Raman spectroscopy based sensitive and specific detection of glycated hemoglobin. Anal Chem. 2012;84:2474–2482.
10. Silveira L, Borges RdCF, Navarro RS, Giana HE, Zângaro RA, Pacheco MTT, et al. Quantifying glucose and lipid components in human serum by Raman spectroscopy and multivariate statistics. Lasers Med Sci. 2017; p. 1–9.
11. Raju B, Rajanikanth A, Bansal C. Silver nanocluster films for glucose sensing by Surface Enhanced Raman Scattering (SERS). Sensing and Bio-Sensing Research. 2016;9:13–16.
12. Shah SH, Svetkey LP, Newgard CB. Branching out for detection of type 2 diabetes. Cell metabolism. 2011;13(5):491–492.
13. Giesbertz P, Daniel H. Branched-chain amino acids as biomarkers in diabetes. Curr Opin Clin Nutr Metab Care. 2016;19(1):48–54.
14. Chen X, Yang W. Branched-chain amino acids and the association with type 2 diabetes. J Diabetes Invest. 2015;6(4):369–370.

15. Yamakado M, Nagao K, Imaizumi A, Tani M, Toda A, Tanaka T, et al. Plasma free amino acid profiles predict four-year risk of developing diabetes, metabolic syndrome, dyslipidemia, and hypertension in Japanese population. Sci Rep. 2015;5:11918.

16. Ben O O, Zephania B, Justus O, Ronald R. Conductive silver paste smeared glass substrates for label-free Raman spectroscopic detection of HIV-1 and HIV-1 p24 antigen in blood plasma. Anal Bioanal Chem. 2017;.

17. Delbanco AS, Burgess ND, Cuni-Sanchez A. Medicinal Plant Trade in Northern Kenya: Economic Importance, Uses, and Origin1. Econ Bot. 2017; p. 1–19.

18. Behera T. Division of Vegetable Science, Indian Agricultural Research Institute, New Delhi 110012, India e-mail: tusar@ iari. res. in. Wild Crop Relatives: Genomic and Breeding Resources: Vegetables. 2011; p. 217.

19. Dormandy JA, Charbonnel B, Eckland DJ, Erdmann E, Massi-Benedetti M, Moules IK, et al. Secondary prevention of macrovascular events in patients with type 2 diabetes in the PROactive Study (PROspective pioglitAzone Clinical Trial In macroVascular Events): a randomised controlled trial. The Lancet. 2005;366(9493):1279–1289.

20. Belfort R, Harrison SA, Brown K, Darland C, Finch J, Hardies J, et al. A placebo-controlled trial of pioglitazone in subjects with nonalcoholic steatohepatitis. N Engl J Med. 2006;355(22):2297–2307.

21. Charan J, Biswas T, et al. How to calculate sample size for different study designs in medical research? Indian journal of psychological medicine. 2013;35(2):121.

22. Szkudelski T. The mechanism of alloxan and streptozotocin action in B cells of the rat pancreas. Physiol Res. 2001;50(6):537–546.

23. Lenzen S. The mechanisms of alloxan-and streptozotocin-induced diabetes. Diabetologia. 2008;51(2):216–226.

24. Zhao J, Lui H, McLean DI, Zeng H. Automated autofluorescence background subtraction algorithm for biomedical Raman spectroscopy. Appl Spectrosc. 2007;61(11):1225–1232.

25. Guangyong Z, Xian Z, Qi F, Xueliang W. Raman spectra of amino acids and their aqueous solutions. Spectrochim Acta, Part A. 2011;78:1187–1195.

26. Zhou G, Yu D, Li S, Yang D. Surface enhanced Raman spectroscopy of leucine and isoleucine. Acta Chim Sin (Chin Ed). 2007;65(7):640.

27. Amanda LJ, Richard AL, Timothy BW. Characterization of amino acids using Raman spectroscopy. Spectrochim Acta, Part A. 2005;61:1585–1594.

28. Belén H, Fernando P, Mama N, Mahmoud G. Vibrational Analysis of Amino Acids and Short Peptides in Hydrated Media. IV. Amino Acids with Hydrophobic Side Chains: L -Alanine, L -Valine, and L -Isoleucine. J Phys Chem B. 2009;113:3169–3178.

29. Almeida FM, Freire PTC, Lima RJC, Remédios CMR, J Mendes F, Melo FEA. Raman spectra of L -isoleucine crystals. J Raman Spectrosc. 2006;37:1296–1301.

30. Yoon MS. The Emerging Role of Branched-Chain Amino Acids in Insulin Resistance and Metabolism. Nutrients. 2016;8(7):405.

31. Lynch CJ, Adams SH. Branched-chain amino acids in metabolic signalling and insulin resistance. Nat Rev Endocrinol. 2014;10(12):723–736.

32. Dieing T, Hollricher O, Toporski J. Confocal raman microscopy. vol. 158. Springer Science & Business Media; 2011.

33. Robitaille L, Hoffer LJ. Measurement of branched chain amino acids in blood plasma by high performance liquid chromatography. Can J Physiol Pharmacol. 1988;66(5):613–617.
